# Circadian regulation of mouse suprachiasmatic nuclei neuronal states shapes responses to orexin

**DOI:** 10.1111/ejn.13506

**Published:** 2017-01-09

**Authors:** Mino D. C. Belle, Hugh D. Piggins

**Affiliations:** ^1^ Faculty of Biology, Medicine, and HealthUniversity of ManchesterAV Hill BuildingManchesterM13 9PTUK

**Keywords:** arousal, electrophysiology, GABA, *Per1*, suprachiasmatic nuclei

## Abstract

Our knowledge of how circadian and homeostatic brain circuits interact to temporally organize physiology and behavior is limited. Progress has been made with the determination that lateral hypothalamic orexin (OXA) neurons control arousal and appetitive states, while suprachiasmatic nuclei (SCN) neurons function as the master circadian clock. During the day, SCN neurons exhibit heterogeneity in spontaneous resting membrane potential (RMP), with some neurons becoming severely depolarized (hyperexcited) and ceasing to fire action potentials (APs), while other neurons rest at moderate RMP and fire APs. Intriguingly, the day phase is when the SCN clock is most readily influenced by arousal, but it is unclear if and how heterogeneity in the excitability state of SCN neurons shapes their response to arousal signals, such as OXA. In whole‐cell recordings we show that during the day OXA recruits GABA‐GABA_A_ receptor signaling to suppress the RMP of hyperexcited silent as well as moderately hyperpolarized AP‐firing SCN neurons. In the AP‐firing neurons, OXA hyperpolarized and silenced these SCN cells, while in the hyperexcited silent neurons OXA suppressed the RMP of these cells and evoked either AP‐firing, depolarized low‐amplitude membrane oscillations, or continued silence at a reduced RMP. These results demonstrate how the resting state of SCN neurons determines their response to OXA, and illustrate that the inhibitory action of this neurochemical correlate of arousal can trigger paradoxical AP firing.

## Introduction

Circadian and homeostatic signals interact to orchestrate the temporal architecture of behavioral and metabolic states (Bechtold & Loudon, 2013). The arousal‐promoting orexin/hypocretin neurons in the lateral hypothalamus and the circadian clock neurons within the suprachiasmatic nuclei (SCN) are two circuits involved in this process. In the SCN, activity of the circadian molecular clock, of which the *Period1* (*Per1*) gene forms a key component, is synchronized by the environmental light‐dark cycle through the glutamatergic retino‐hypothalamic tract. This drives daily changes in electrical states, with SCN neurons being overtly more active (upstate) across the day and less excited (downstate) at night (Brown & Piggins, 2007; Colwell, 2011; Belle, 2015). Such pronounced day‐night variation in SCN electrical activity functions to communicate temporal signals to the rest of the brain and body, including orexin neurons. Indeed, the activity of orexin neurons is under circadian control (Zhang *et al*., 2004), with their molecular activity (Estabrooke *et al*., 2001; Marston *et al*., 2008) and orexin release elevated during the behaviorally active circadian night in nocturnal rodents (Deboer *et al*., 2004).

The phase of the SCN circadian clock is sensitive to feedback from arousal‐promoting stimuli, particularly during the day (Mistlberger & Antle, 2011; Hughes & Piggins, 2012). Such stimuli activate orexin neurons (Estabrooke *et al*., 2001; Marston *et al*., 2008; Webb *et al*., 2008), whose brain‐wide projection targets include several structures of the neural circadian system (McGranaghan & Piggins, 2001; Backberg *et al*., 2002). Indeed, orexin neurons form putative synaptic appositions onto SCN clock neurons and transcripts for orexin receptor 1 and 2 (OX_1_ and OX_2_, respectively) are expressed in the SCN (Belle *et al*., 2014). Orexin exists as two forms, orexin‐A (OXA) and orexin‐B, and exogenously applied OXA acts to mainly suppress electrical activity of rodent SCN cells (Brown *et al*., 2008; Klisch *et al*., 2009; Belle *et al*., 2014). This suggests that OXA released in the vicinity of SCN neurons during states of arousal can influence excitability in this brain region.

The SCN neural population is both molecularly and neurochemically heterogeneous. For example, in mice in which an enhanced green fluorescent protein (EGFP) reports the activity of the *Per1* promotor, many SCN neurons express the *Per1*‐driven construct (*Per1*‐EGFP+ve), but in some neurons this construct is not detected (EGFP−ve, presumed non‐*Per1* cells) (Kuhlman *et al*., 2000; Belle *et al*., 2009). This heterogeneity also extends to the neurophysiology of SCN neurons during the day, but not at night. Indeed, targeted whole‐cell recordings in mouse SCN revealed that although the intrinsic membrane excitability states of *Per1‐*EGFP+ve neurons and EGFP−ve cells are generally similar across much of the day‐night cycle, during the mid‐afternoon (ZT6‐10) they are vastly different (Belle *et al*., 2009). During the mid‐afternoon, *Per1‐*EGFP+ve neurons enter hyperexcited electrical states, where their resting membrane potential (RMP) becomes severely depolarized (~ −33 mV) and their input resistance (*R*_input_) elevates to maximal values (~ 2–3 GΩ) (Belle *et al*., 2009). Consequently, at these hyperexcited states *Per1‐*EGFP+ve neurons stop generating action potentials (APs) through depolarization blockade, and become completely silent or generate depolarized low‐amplitude membrane oscillations (DLAMOs; Belle *et al*., 2009; Diekman *et al*., 2013). By stark contrast, EGFP−ve neurons do not enter hyperexcited states at any time across the day‐night cycle, and in the mid‐afternoon they exhibit lower *R*_input_ (~ 1.5 GΩ) and rest at moderate RMP (~ −45 mV) where they can readily discharge APs (Belle *et al*., 2009).

The physiological significance for this dichotomy in *Per1*‐EGFP+ve and EGFP−ve excitability states during this time of the day remains elusive, and here we explore the possibility that their resting states shape and influence the way SCN neurons respond to inputs, specifically the actions of the arousal‐evoking neuropeptide, OXA.

## Materials and methods

### Animals

We used 28 male and female mice (8 weeks to 6 months old) hemizygous for the *Period1*::d2EGFP transgene (*Per1*‐EGFP‐expressing mice in which an enhanced destabilized green fluorescent protein (EGFP) reports the activity of the *Per1* promoter (a gift from Professor D. McMahon, Vanderbilt University, USA; see Kuhlman *et al*., 2000). These animals were bred and supplied by the Biological Services Facility of the University of Manchester, and were group‐housed on a 12 hour light : 12 hour dark (L : D) cycle (lights on at 07:00). Food and water were available *ad libitum*. All experimental procedures were carried out according to the provisions of the UK Animal (Scientific Procedures) Act 1986, and approved by the Research Ethics Committee of the University of Manchester.

### Preparation of living mouse SCN brain slices for *in vitro* whole‐cell recordings

Brain sections were prepared during the subjective day at Zeitgeber Time (ZT) 1–2, with ZT0 defined as the time of lights on. Coronal brain slices containing the mid‐level of the rostro‐caudal SCN were prepared from 28 male and female *Per1*‐EGFP mice (one slice per animal), as previously described (Belle *et al*., 2009, 2014). Briefly, animals were deeply anesthetized with isoflurane to minimize pain and discomfort and killed by cervical dislocation and decapitation. Brains were immediately removed and 250 μm thick coronal slices cut with a vibroslicer (Campden Instruments, Loughborough, UK) in an ice‐cold (4 °C) low Na^+^/Ca^2+^, high Mg^2+^ sucrose‐based artificial cerebrospinal fluid (aCSF) (in mm: NaCl 95; KCl 1.8; KH_2_PO_4_ 1.2; CaCl_2_ 0.5; MgSO_4_ 7; NaHCO_3_ 26; glucose 15; sucrose 50; Phenol Red 0.005 mg/L; oxygenated with 95% O_2_; 5% CO_2_; pH 7.4, measured osmolarity 300–310 mOsmol/kg). For whole‐cell patch‐clamp recordings, slices were transferred to a recording chamber mounted on the stage of an upright Olympus epi‐fluorescence microscope (BX51WI; Olympus, Japan), where they were continuously perfused (~ 3 mL/min) with recording aCSF. The composition of the recording aCSF was identical to the incubation solution except for the following (mm): NaCl 127; CaCl_2_ 2.4; MgSO_4_ 1.3; sucrose 0.

### Whole‐cell recordings

**Figure 1:**
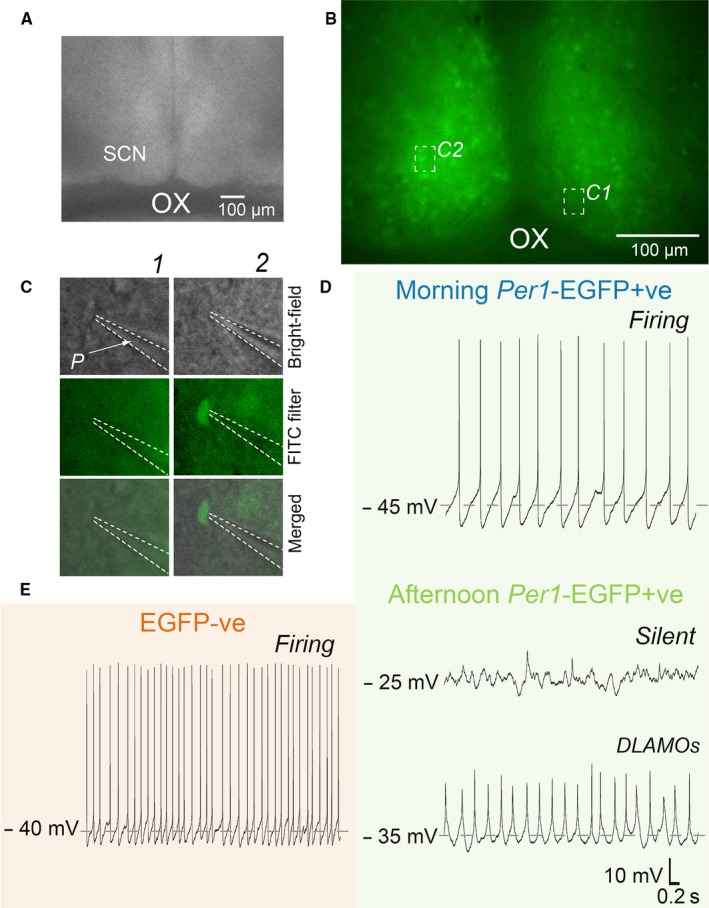
Whole‐cell targeting of *Per1*‐EGFP+ve and EGFP‐ve neurons in a living SCN slice. (A) Recording setup showing bright‐field image (4 ×) of a living coronal SCN slice (250 μm thick) taken at the mid‐rostro‐caudal level. (B) Image of *Per1*‐EGFP+ve fluorescence signal (20 ×) taken from the slice shown in (A) with a FITC (488 nm, optimum for EGFP) filter. Insets *C1* and *C2* are representative photomicrographs showing patch pipette targeting of EGFP−ve (*C1*, follow vertically) or *Per1*‐EGFP+ve (*C2*) neurons (40 ×) in the slice shown in A and B. Insets rows show bright‐field, fluorescence (FITC), and merged bright‐field and fluorescence images. (D and E) Spontaneous membrane excitability states of *Per1*‐EGFP+ve (D) and EGFP−ve (E) SCN neurons over the day. (D) Example of typical current‐clamp traces from *Per1*‐EGFP+ve SCN neurons recorded in the morning (ZT2–5) and afternoon (ZT6–10). Note that *Per1*‐EGFP+ve neurons recorded in the morning are at moderate resting membrane potential (RMP: ~ −45 mV) and discharging action potentials (APs), while in the afternoon they become hyperexcited with RMPs of −25 to −35 mV and unable to generate APs. These cells are either silent or generating depolarized low‐amplitude membrane oscillations (DLAMOs). By contrast, EGFP−ve neurons (E) remain at moderate RMP throughout the day, firing APs. Dashed lines in C indicate the outline of the patch pipette. SCN, suprachiasmatic nuclei; OX, optic chiasm; *P*, patch pipette. [Colour figure can be viewed at wileyonlinelibrary.com].

Targeted recordings (current‐ or voltage‐clamp) were made from *Per1*‐EGFP positive (henceforth referred to as *Per1*‐EGFP+ve) neurons and cells in which EGFP could not be detected (EGFP−ve), in the ventral and central sub‐regions of the SCN on the coronal plane. These SCN anatomical regions were chosen as they are associated with arousal‐promoting signal input to the SCN (Morin, 2013). *Per1*‐EGFP+ve neurons were visually identified and distinguished from EGFP−ve cells with a 40 × water immersion UV objective (LUMplanFL/IR; Olympus) using epifluorescence with a Leica camera (DFC 350 FX) and capturing software (Leica Application Suite; Leica Microsystems, UK), as previously described (Belle *et al*., 2009, 2014; Scott *et al*., 2010; see also Fig. 1). Giga‐ohm seal and cell membrane rupture were done under infra‐red video‐enhanced differential interference contrast microscopy with an infra‐red camera (Hitachi, Japan). An Axoclamp 2A amplifier (Molecular Devices, CA, USA) was used for current‐clamp recordings as previously described (Belle *et al*., 2009, 2014). Patch pipette electrodes (7–10 MΩ) were fashioned from thick‐walled borosilicate glass capillaries (Harvard Apparatus) using a two‐stage vertical micropipette puller (PB‐10; Narashige, Japan). Unless otherwise stated, electrodes were filled with an internal solution containing (mm: K‐gluconate 130; KCl 10; MgCl_2_ 2; K_2_‐ATP 2; Na‐GTP 0.5; Hepes 10, EGTA 0.5; pH adjusted to 7.3 with KOH, measured osmolarity 295–300 mOsmol/kg). Pipette series resistance (typically 10–30 MΩ) was corrected using bridge‐balance in current‐clamp experiments and was not compensated during voltage‐clamp recordings. Cells were accepted for analysis only if the series resistance was below 30 MΩ, remained within 20% of this value throughout the recordings and the RMP remained stable for over 2 min before orexin‐A (OXA) application. RMP, spontaneous firing rate (SFR) and input resistance (*R*_input_) were determined within 1–2 min of membrane rupture. The average firing rate in firing cells was measured as the number of spikes per second within a 20 s window of stable firing, and average RMP was measured as the mean voltage (including AP or DLAMOs, for cells displaying such activities) over a 20 s window using a custom‐written SPIKE2 script [Cambridge Electronic Design (CED), Cambridge, UK]. *R*_input_ was assessed only in current‐clamp recordings and was estimated by a series of hyperpolarizing current pulses (−10 to −30 pA for 500 ms). Signals were sampled at 30 kHz, and stored and analyzed on a personal computer using spike2 software (Version 7; CED). All data acquisition and step protocols were generated through a micro 1401 mkII interface (CED).

#### Synaptic current measurement

Post‐synaptic currents (PSCs) were measured with an Axopatch 200B amplifier (Molecular Devices), holding the cells at −70 mV. Patch pipettes (4–6 MΩ) were fashioned as described above and filled with an internal solution that was identical to the one used in current‐clamp recordings, except for the following (mm): K‐gluconate 120; KCl 20. Dialyzing SCN neurons with this concentration of Cl^−^ in the pipette causes inhibitory PSCs to reverse between −40 to −50 mV (Itri & Colwell, 2003; Belle *et al*., 2014). At −70 mV this positive shift in Cl^−^ reversal potential causes GABA and glutamate PSCs to appear as inward currents. To discriminate between excitatory and inhibitory PSCs, a cocktail of antagonists (AP5 and CNQX) for the glutamate and/or GABA_A_ (Gabazine) receptors were used.

### Drugs and analysis

Stock solutions (1000 × concentration of working dilutions) for orexin‐A (OXA), D‐2‐amino‐5‐phosphonopentanoate (AP5), 6‐cyano‐7‐nitroquinoxaline‐2,3‐dione (CNQX) and gabazine (Tocris, Bristol, UK) were made in purified MilliQ water. Working drug dilutions were made in aCSF immediately before their application. Recording aCSF (drug‐free or containing drugs) solution was bath applied to SCN slices by gravity‐feed perfusion. Switching from drug‐free to drug‐containing aCSF was done using an in‐house built drug‐switching solenoid valves system. All recording and pharmacology methods used for isolating and measuring the effects of OXA on synaptic activity, RMP and AP firing frequency in SCN neurons were as previously established (Belle *et al*., 2014). Analysis of PSC frequency and amplitude were conducted offline by template‐based sorting in clampfit 10.2 (Molecular Devices). Synaptic events were detected with an amplitude threshold of 5 pA (see Belle *et al*., 2014).

Grouped data from *Per1*‐EGFP+ve and EGFP−ve neurons were initially examined for normality using the Kolmogorov–Smirnov (K–S) test. When normality was found, data were statistically compared using two‐way anova with repeated measures and/or paired/unpaired two‐tailed *t* tests. Bonferroni correction was used for multiple comparisons. In the absence of normality, statistical comparisons were made using the Mann–Whitney *U* Test or related samples Wilcoxon Test (spss version 22; SPSS Inc., Chicago, IL, USA). For all tests, *P *<* *0.05 was considered significant. All numerical data both in text and graphs represent mean ± SEM.

## Results

### Mid‐afternoon *Per1*‐EGFP+ve and EGFP−ve neurons differ in their excitability states

We used epifluorescence and during the afternoon (ZT6‐10) made targeted whole‐cell current‐clamp recordings from identified *Per1‐*EGFP+ve and EGFP−ve neurons (*n* = 21 neurons each, from 14 animals) in the ventral and central SCN sub‐regions to determine if and how their intrinsic excitability states contributed to their response to OXA (see Materials and methods and Fig. 1). Using this approach we confirmed previous results (Belle *et al*., 2009) that during the mid‐afternoon *Per1‐*EGFP+ve neurons enter hyperexcited electrical states (Fig. 1D) with RMP and *R*_input_ values (RMP = −30.5 ± 0.8 mV; *R*_input_ = 2.4 ± 0.1 GΩ) that are significantly higher than those measured in EGFP−ve cells (RMP = −45 ± 1 mV; *T*_(40)_ = 11.393, *P* < 0.0001; *R*_input_ = 1.4 ± 0.05 GΩ : *Z* = −5.472, *P* < 0.0001). As *Per1‐*EGFP+ve neurons in these electrical states cannot discharge APs, while EGFP−ve cells can, comparison of SFR revealed a significant difference, with elevated SFR values in EGFP−ve neurons (Fig. 1E) and no spiking activity in the severely depolarized *Per1‐*EGFP+ve cells (*Per1‐*EGFP+ve = 0 Hz*;* EGFP−ve = 4 ± 0.4 Hz; *Z* = −5.946, *P *<* *0.0001: Fig. 1D).

### OXA suppresses the RMP of *Per1*‐EGFP+ve and EGFP−ve neurons

During the day, OXA acts in the SCN to mainly suppress excitability in *Per1*‐EGFP+ve neurons by elevating the pre‐synaptic release of GABA onto post‐synaptic GABA_A_ receptors (Belle *et al*., 2014). To test if a similar mechanism of OXA suppression operates in EGFP−ve cells and to investigate whether the contrasting electrical states in mid‐afternoon *Per1*‐EGFP+ve and EGFP−ve neurons functionally determine their responses to OXA, we bath applied OXA (80 nm for 3 min) to the current‐clamped *Per1‐*EGFP+ve and EGFP−ve neurons, and compared if and how their electrical states changed in the presence of this neuropeptide.

**Figure 2:**
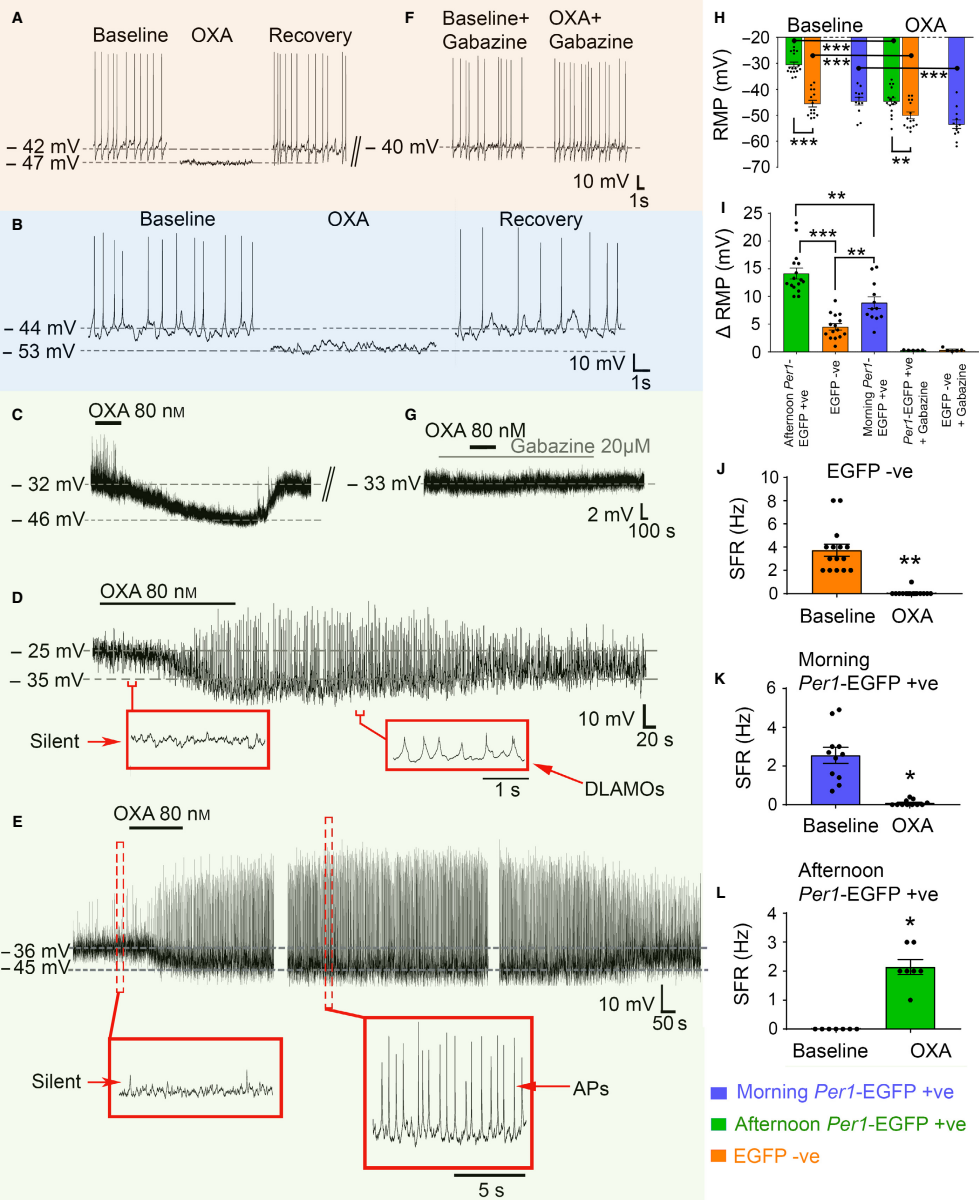
Orexin (OXA) indirectly suppresses the activity of *Per1*‐EGFP+ve and EGFP−ve SCN neurons. (A) Typical inhibitory response of an SCN EGFP−ve neuron to bath application of OXA during the mid‐afternoon, causing membrane hyperpolarization and complete suppression of AP firing. (B–E) Whole‐cell recordings showing suppressive response of moderately hyperpolarized/excited morning (B) and hyperexcited afternoon (C–E) *Per1*‐EGFP+ve neurons to bath applied OXA. In morning cells, OXA caused membrane hyperpolarization and suppression of AP firing. In some afternoon cells the OXA‐evoked RMP suppression triggered paradoxical firing of APs (E, 7/16 responsive cells; see L), while in others it elicits DLAMOs activity (D, 4/16 responsive cells). In the remaining OXA responsive neurons (5/16) RMP hyperpolarization did not evoke APs or DLAMOs and the cells remained silent (C). (F–G) Consecutive traces from cells (A) and (C), respectively, showing examples that co‐treatment of cells suppressed by OXA (e.g. A–C) with OXA and the selective GABA_A_ receptor antagonist, gabazine, abolished OXA effects. (H–L) Summary analyses of OXA's effects on excitability of *Per1*‐EGFP+ve and EGFP−ve neurons, with (H) showing suppression of RMP, which in both populations was significantly different from baseline (12 morning and 16 afternoon *Per1*‐EGFP+ve, and 15 EGFP−ve cells). (I) The magnitude of this OXA‐evoked change (Δ) in RMP was significantly different when cell‐type and cell‐state were compared; hyperexcited afternoon *Per1*‐EGFP+ve > moderately resting morning *Per1*‐EGFP+ve > EGFP−ve neurons. Co‐application of gabazine with OXA prevented Δ in RMP in all cells tested (*n* = 5 *Per1*‐EGFP+ve and 5 EGFP−ve cells; single degree of freedom *t*‐test; *P* > 0.05). (J–K) In EGFP−ve and morning *Per1*‐EGFP+ve neurons OXA‐evoked hyperpolarization significantly suppressed firing activity. (L) Quantification of the paradoxically‐elicited spiking activity in *Per1*‐EGFP+ve cells during OXA‐evoked hyperpolarization. ***P < 0.0001; **P < 0.01; *P < 0.05. Data shown in panels H to L are plotted as mean ± SEM, with data points from individual neurons depicted as filled black circles. [Colour figure can be viewed at wileyonlinelibrary.com].

The majority of *Per1*‐EGFP+ve (16/21; ~ 76%) and EGFP−ve (16/21; ~ 76%) neurons tested responded to OXA, with a ≥ 2 mV change in their RMP. Within these OXA‐sensitive cells, most (15/16; ~ 94%) of EGFP−ve neurons responded by significant hyperpolarization of their RMP when compared with baseline values (*T*_(14)_ = 7.931, *P *<* *0.0001; Fig. 2A and H), and only one cell showed membrane depolarization (data not shown). The hyperexcited *Per1*‐EGFP+ve neurons (16/16) also responded to OXA by suppressing their RMP to levels that were significantly different from baseline (*T*_(15)_ = 14.626, *P *<* *0.0001; Fig. 2C–E and H). By comparison, application of OXA in the presence of the selective OXA receptor antagonist, SB334867 (10 μm), prevented all these cells from changing their RMP (data not shown), replicating our previous observation (Belle *et al*., 2014). Subsequent two‐way repeated measures anova comparison revealed a significant main effect of cell‐type (*F*_1, 29_ = 39.793, *P* < 0.0001), treatment (*F*_1, 29_ = 267.252, *P* < 0.0001) and cell‐type × treatment interaction (*F*_1,29_ = 71.149, *P* < 0.0001) in RMP. Comparisons of RMPs between these two OXA‐responsive cell‐types showed that, as in the overall cell populations, *Per1*‐EGFP+ve neurons rested at a significantly more depolarized level than EGFP−ve cells (−30.5 ± 1 vs. −45.5 ± 1.3 mV; *T*_(29) _= 9.423, *P *<* *0.0001). The RMPs attained by *Per1*‐EGFP+ve and EGFP−ve cells during their maximal steady‐state OXA response were also significantly different (−44.6 ± 1.3 vs. −50 ± 1.3 mV; *T*_(29)_ = 2.950, *P *=* *0.006; Fig. 2H). When delta RMP between the two cell populations was compared, the magnitude of OXA‐evoked hyperpolarization in *Per1*‐EGFP+ve neurons (*n* = 16; 14.2 ± 1 mV) was significantly larger (*T*_(29)_ = 8.435, *P* < 0.0001) than those measured in EGFP−ve cells (*n* = 15; 4.5 ± 0.6 mV; Fig. 2I).

### Cell‐state and cell‐type determine the response magnitude of SCN neurons to OXA

The above observation suggests that afternoon cell‐state and/or cell‐type underpins the contrasting magnitude in response to OXA. To directly investigate these possibilities we next assessed the magnitude of OXA‐evoked membrane suppression in *Per1*‐EGFP+ve cells current‐clamped in the morning (ZT2‐5), a time when the natural intrinsic excitability state of *Per1*‐EGFP+ve neurons is moderate and similar to that measured in EGFP−ve cells (Fig. 1D, compare with 1E), and importantly, is vastly different from afternoon *Per1*‐EGFP+ve cells (Fig. 1D, see also Belle *et al*., 2009). We compared the magnitude of these morning responses to OXA with those measured in afternoon neurons. As previously described, morning *Per1*‐EGFP+ve neurons rested at moderate RMPs (−44.6 ± 1.5 vs. afternoon −30.5 ± 1 mV; *T*_(26)_ = −8.301, *P *<* *0.0001) and can discharge APs (2.5 ± 0.4 vs. 0 Hz; *Z* = −3.594, *P* < 0.0001; Fig. 1D, 17 neurons from six animals). When challenged with OXA, the majority of these cells (12/17; 71%) reduced excitability by hyperpolarization and suppression or abolishment of AP firing (−44.6 ± 1.5 vs. −53.4 ± 1.7 mV, *T*_(11)_ = 8.380, *P *<* *0.0001; 2.6 ± 0.4 vs. 0.1 ± 0.04 Hz, *Z* = −2.936, *P* = 0.003; Fig. 2B, H and K), while 2/17 (~ 12%) did not respond and 3/17 (~ 17%) showed membrane excitation (data not shown). When the magnitude of this RMP change was compared with that measured in hyperexcited afternoon *Per1*‐EGFP+ve neurons, a significant difference was detected (8.9 ± 1.1 vs. 14.2 ± 1 mV; *T*_(26)_ = −3.654, *P *=* *0.001), indicating that OXA evoked larger delta RMP in afternoon than in morning *Per1*‐EGFP+ve cells (Fig. 2I). We next compared OXA‐evoked delta RMP measured in morning *Per1*‐EGFP+ve with that assessed in EGFP−ve cells to test whether elements of cell‐type with similar excitability state also determine the magnitude of OXA response. Interestingly, although the steady‐state RMP attained during OXA application was not significantly different in these two cell populations (−53.4 ± 1.7 vs. −50 ± 1.3 mV; *P* > 0.05; Fig. 2H), delta RMP in *Per1*‐EGFP+ve neurons was significantly larger than that measured in EGFP−ve cells (8.9 ± 1.1 vs. 4.5 ± 0.6 mV; *T*_(25)_ = 3.822, *P *=* *0.001; Fig. 2I). It is noteworthy, however, that when the magnitude of the T‐values and mean difference between the deltas were considered across time of day (morning vs. afternoon) as well as neuronal type (*Per1*‐EGFP+ve vs. EGFP−ve), cell‐state rather than cell‐type emerged as the more defining determinant of delta RMP magnitude to OXA (ΔRMP in afternoon *Per1*‐EGFP+ve > morning *Per1*‐EGFP+ve > EGFP−ve; Fig. 2I).

### OXA‐evoked suppression of RMP elicits distinct membrane excitability activities in afternoon *Per1*‐EGFP+ve and EGFP−ve neurons

Intriguingly, OXA‐evoked hyperpolarization produced diverse changes in afternoon *Per1*‐EGFP+ve membrane excitability that were distinct from those seen in EGFP−ve cells (*n* = 16 and 16 cells, respectively, from 14 animals). In EGFP−ve neurons, OXA‐evoked membrane hyperpolarization caused significant suppression or abolishment of AP generation (3.7 ± 0.5 Hz vs. 0.07 ± 0.07 Hz; *Z* = −3.431, *P* = 0.001; Fig. 2A and J), effects that were comparable to those elicited in morning *Per1*‐EGFP+ve neurons (see above). By comparison, membrane hyperpolarization by OXA in afternoon *Per1*‐EGFP+ve neurons produced three distinct excitability activities. In some of these severely depolarized silent cells (5/16; ~ 31%), the membrane hyperpolarized but the cells remained silent (Fig. 2C). In others (4/16; 25%), OXA‐evoked RMP suppression caused these cells to generate DLAMOs (Fig. 2D). Unexpectedly, in the remaining cells (7/16; ~ 44%), suppression of RMP by OXA paradoxically triggered spiking activity (2.1 ± 0.3 Hz; Fig. 2E and L), presumably by transiting these cells from depolarization blockade (~ −25 to −36 mV) to values of RMP (~ −45 mV) at which they can generate APs. This paradoxical increase in firing frequency during OXA‐evoked hyperpolarization was significant when compared with their initial baseline silent state (*Z* = −2.414, *P* = 0.016; Fig. 2L).

**Figure 3:**
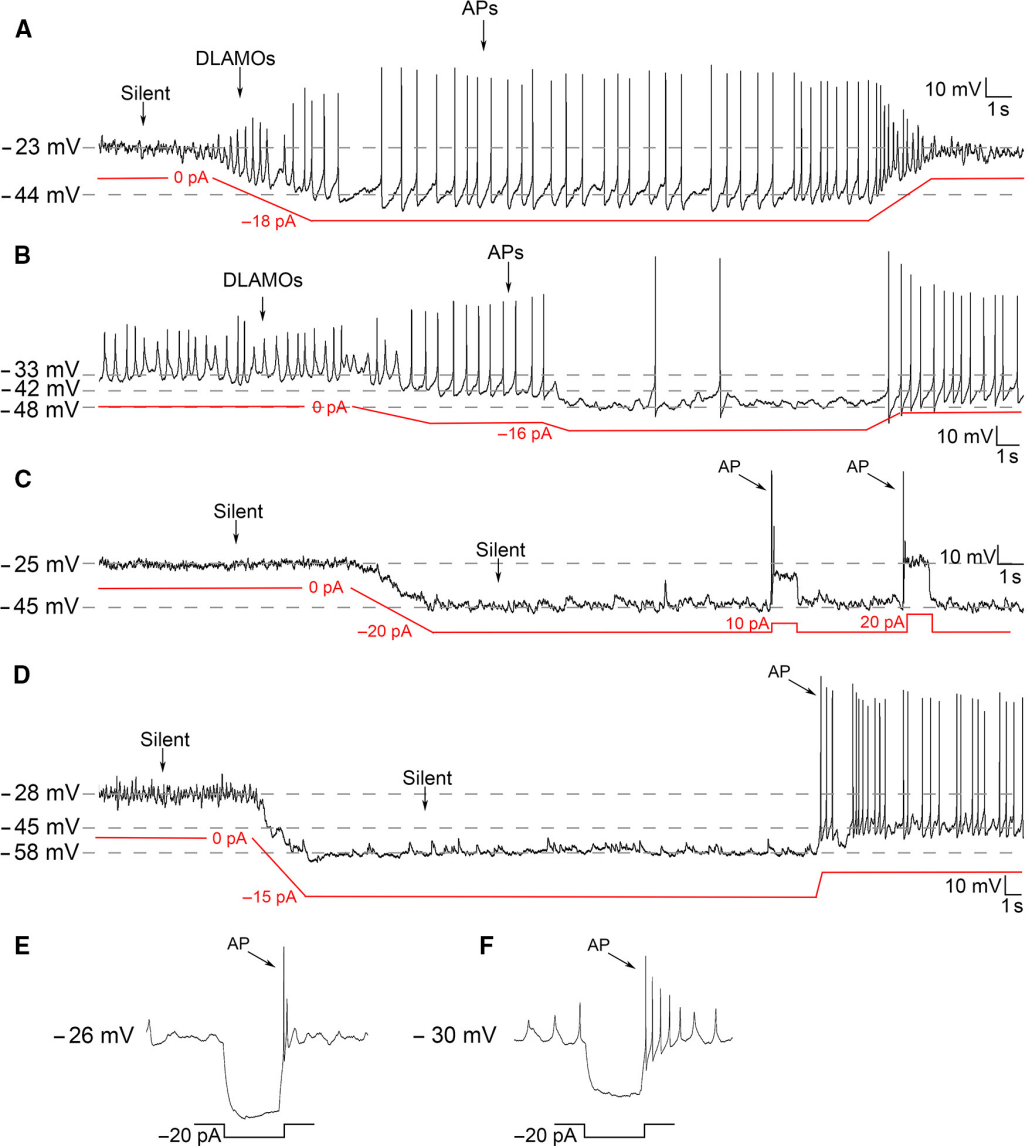
Manual hyperpolarization elicits a range of electrical states in hyperexcited *Per1*‐EGFP+ve neurons that are comparable to those evoked by OXA. (A) Typical silent *Per1*‐EGFP+ve neuron firing APs when manually maintained at ~ −44 mV and producing DLAMOs when transiting between silent and firing states (equivalent to OXA‐evoked responses in Fig. 2E). Gradual release from forced hyperpolarization returned this cell to DLAMO and silent states. (B) A DLAMOs producing neuron forced to fire APs upon manual hyperpolarization and stopped/reduced AP firing when RMP is maintained at a more negative potential. Cell discharges APs when RMP is returned to firing range. (C) Typical silent *Per1*‐EGFP+ve neuron that sustained silence when hyperpolarized to a RMP that promotes AP discharge (equivalent to OXA‐evoked response shown in 2C). Depolarizing pulses of 10 and 20 pA elicited AP firing, demonstrating this cell's ability to produce an AP when acutely excited. (D) Silent hyperexcited *Per1*‐EGFP+ve neuron producing APs when maintained at the firing range (~ −45 mV) but in between was rendered silent by RMP maintenance below (−58 mV) firing threshold. (E and F) Typical rebound spike response in depolarized silent cell by a hyperpolarizing pulse which transiently removes the cell from depolarization blockade, re‐activating an AP. [Colour figure can be viewed at wileyonlinelibrary.com].

The trigger for the range of excitability activities seen in afternoon *Per1*‐EGFP+ve neurons by OXA could be simply by the relief of these cells from depolarization blockade through suppressive GABA‐GABA_A_ signaling. We therefore tested whether such a common and general mechanism of OXA‐dependent rescue of hyperexcited *Per1*‐EGFP+ve neurons from depolarization blockade accounts for the array of electrical behaviors seen during their responses to OXA. To do this, we recorded from afternoon *Per1*‐EGFP+ve neurons and mimicked the hyperpolarizing effects of OXA by applying controlled steady‐state negative currents which artificially hyperpolarized and maintained their RMPs at appropriate levels (from −25 to ~ −35 and −45 mV; Fig. 3). From the 10 cells actively manipulated in this way, 7/10 (70%, from three animals) fired APs when maintained at ~ −45 mV (Fig. 3A, B and D). Of these seven cells, 3 (43%) produced DLAMOs when membrane potential was maintained at ~ −33 mV (Fig. 3A); observations that support our previous finding (Belle *et al*., 2009). The remaining cells (3/10; 30%) hyperpolarized but sustained silence throughout steady‐state current injection (from −25 to −45 mV; Fig. 3C). Passage of a square depolarizing pulse during manual suppression produced APs in these neurons (Fig. 3C). This suggests that intrinsic mechanisms operate to maintain membrane silence in these cells even in the potential range at which APs could be spontaneously fired, as shown by the acute excitatory stimulation. Indeed, this observation supports previous findings that single SCN neurons do not necessarily discharge APs, even when at values of RMP that are permissive of AP firing (M. D. C. Belle and H. D. Piggins, unpublished observations and see also (Rohling *et al*., 2006)). Furthermore, in these hyperexcited cells a rebound spike can be triggered if the RMP is acutely removed from depolarization blockade by a brief hyperpolarizing pulse (Fig. 3E and F). By contrast, manual steady‐state hyperpolarization of morning *Per1*‐EGFP+ve and afternoon EGFP−ve cells invariantly ceased AP generation (data not shown).

### OXA‐evoked increase in spontaneous post‐synaptic current amplitude is significantly larger in afternoon *Per1*‐EGFP+ve neurons than in EGFP‐ve cells

When OXA‐responsive *Per1*‐EGFP+ve (*n* = 5) and EGFP−ve (*n* = 5) neurons were re‐tested with OXA in the presence of the selective GABA_A_ receptor blocker, gabazine, no effects on RMP were observed (Fig. 2F, G, and I). This replicates our earlier finding that gabazine blocks RMP effects of OXA on *Per1*‐EGFP+ve neurons during the day (Belle *et al*., 2014), and extends this to show that gabazine also prevents hyperpolarizing actions of OXA in EGFP−ve cells. This indicates that in both cell types, OXA recruits indirect/pre‐synaptic GABA to signal via the GABA_A_ receptor and hyperpolarize SCN neurons during the day. Our afternoon current‐clamp data also demonstrates that the magnitude of RMP suppression by this pre‐synaptic action of OXA is significantly larger in hyperexcited *Per1*‐EGFP+ve neurons than in moderately resting EGFP−ve cells (Fig. 2I). This suggests that *Per1*‐EGFP+ve and EGFP−ve SCN neurons have differential response characteristics to OXA‐evoked synaptic GABA‐GABA_A_ receptor signaling. To investigate this, we next performed voltage‐clamp recordings in SCN *Per1*‐EGFP+ve and EGFP−ve neurons (*n* = 7 cells each, from five animals) and compared the effects of bath‐applied OXA on the frequency and amplitude of PSCs. To isolate GABAergic events, we performed these recordings in the presence of AP5 and CNQX, respective blockers of NMDA and non‐NMDA receptors.

**Figure 4:**
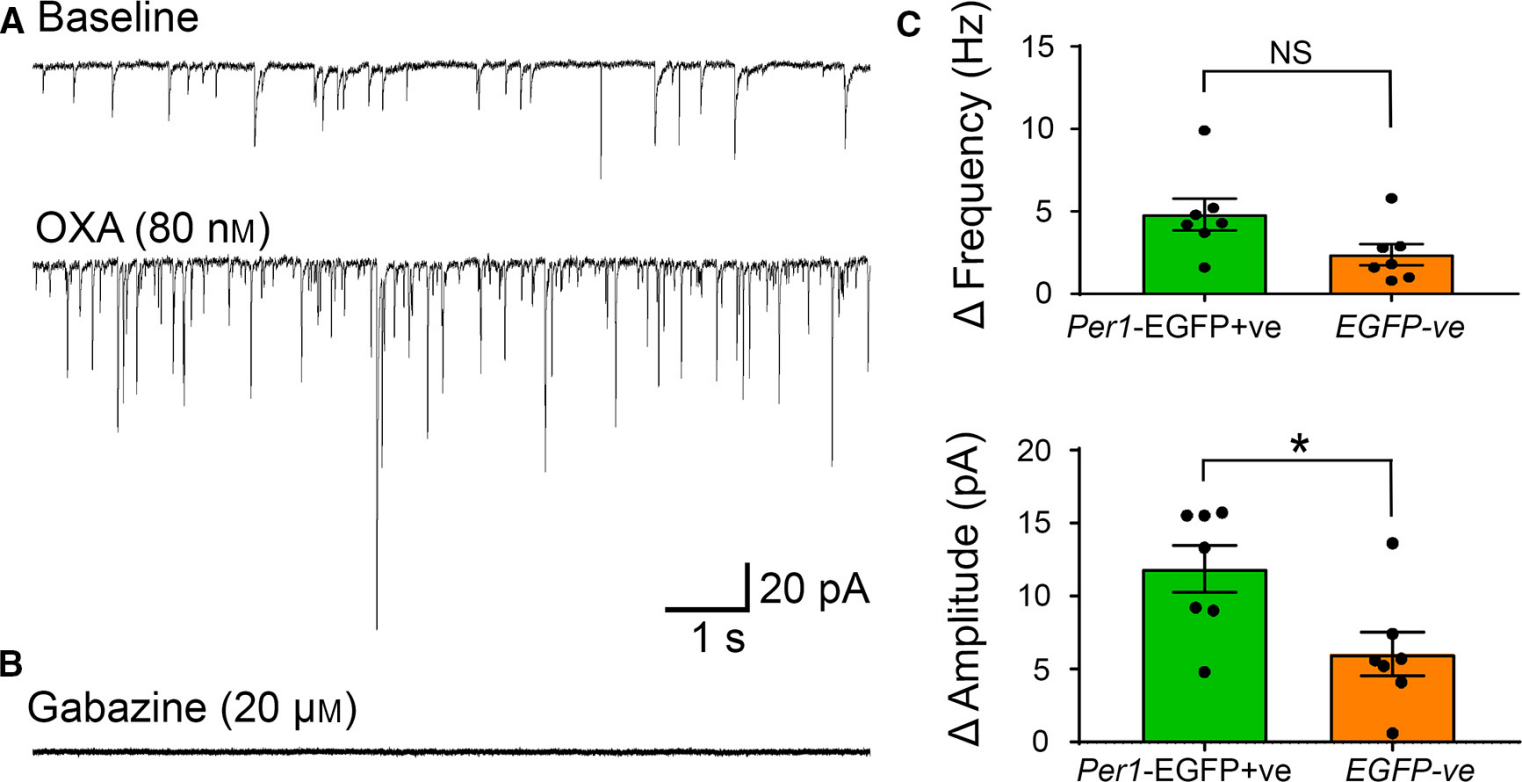
Orexin (OXA) increases the frequency and amplitude of post‐synaptic currents (PSC) in *Per1*‐EGFP+ve and EGFP−ve neurons. (A) Example of traces from a *Per1*‐EGFP+ve neuron (voltage‐clamped at −70 mV) with the top and bottom traces showing PSC frequency in baseline (top) and during OXA (bottom) bath application. This recording was made in the presence of the specific glutamatergic receptor antagonists (AP5 and CNQX, NMDA/AMPA/kainate‐types) in the bath, which did not prevent the OXA‐evoked increase in PSC frequency and amplitude. However, these OXA‐evoked events were completely abolished in the presence of the specific GABA_A_ receptor blocker, gabazine (B). (C) Summary of OXA‐dependent delta increase in PSC frequency and amplitude in *Per1*‐EGFP+ve and EGFP−ve neurons, showing significant differences between cell‐type in PSC amplitude, but not frequency (NS). *P < 0.05. Analysis was performed on recordings from 7 *Per1*‐EGFP+ve and 7 EGFP−ve neurons. Data shown in panel C are plotted as mean ± SEM, with data points from individual neurons depicted as filled black circles. [Colour figure can be viewed at wileyonlinelibrary.com].

Our results indicate that OXA significantly increased the GABAergic PSC frequency (*Per1*‐EGFP+ve baseline = 4.1 ± 0.5 Hz; OXA = 9 ± 0.9 Hz, *T*_(6)_ = −5.158, *P *=* *0.002; and EGFP−ve baseline = 2.3 ± 0.6 Hz; OXA = 4.7 ± 0.7 Hz, *T*_(6)_ = −3.829, *P *=* *0.009) and amplitude (*Per1*‐EGFP+ve baseline = 14.2 ± 1.8 pA; OXA = 26.1 ± 1.2 pA, *T*_(6)_ = −7.437, *P *< 0.0001; and EGFP−ve baseline = 17.5 ± 3.2 pA; OXA = 23.6 ± 3.2 pA, *T*_(6)_ = −4.043, *P *=* *0.007) in ~ 90% of recorded cells (Fig. 4A and C). When comparisons of PSC amplitude and frequency were performed across cell‐types, two‐way repeated measures anova revealed a significant main effect of cell‐type (*F*_1,12_ = 17.787, *P* = 0.001) and treatment (*F*_1,12_ = 41.202, *P* < 0.0001), but no cell‐type × treatment interaction (*F*_1,12_ = 4.400, *P* = 0.058) for frequency. For amplitude, no main effect of cell‐type was seen (*F*_1,12_ = 0.016, *P* = 0.902), but significance in main effect of treatment (*F*_1,12_ = 67.156, *P* < 0.0001) and cell‐type × treatment interaction (*F*_1,12_ = 7.155, *P* = 0.02) were found. Subsequent comparisons of delta amplitude and frequency revealed that the OXA‐evoked change in PSC amplitude was significantly different in these two cell‐types (*T*_(12)_ = 2.659, *P *=* *0.021), with larger PSC measured in *Per1*‐EGFP+ve neurons than in EGFP−ve cells (11.9 ± 1.6 vs. 6 ±1.5 pA; Fig. 4C). Contrary with this, no difference was seen in OXA‐evoked delta frequency in these two cell populations, although this difference approached significance (4.8 ± 1 Hz vs. 2.4 ± 0.6 Hz; *T*_(12)_ = 2.109, *P *=* *0.057). In all recordings, the OXA‐sensitive synaptic events were completely abolished by bath applied gabazine (Fig. 4B), indicating that they were indeed GABA_A_ receptor mediated.

## Discussion

An important quest in neuroscience is identifying processes that shape how neurons respond to inputs. Here, we show that molecular clock‐driven changes in membrane potential and neural state is a key factor in shaping SCN neuronal responses to a neurochemical correlate of arousal. Specifically, we revealed that the intrinsic excitability state of *Per1*‐EGFP+ve and EGFP−ve neurons during the day determines how these cells respond to OXA.

Our results indicate that the vast majority of moderately hyperpolarized AP‐discharging EGFP−ve (~ 94%) and *Per1*‐EGFP+ve (~ 71%) cells were hyperpolarized and silenced by OXA. This differs from hyperexcited *Per1*‐EGFP+ve neurons in which diverse electrical activities were seen during OXA‐evoked RMP suppression, ranging from DLAMO generation to paradoxical spiking. In both cell populations these OXA‐dependent electrical behaviors relied on GABA‐GABA_A_ receptor signaling, but not ionotropic glutamatergic transmission. This is consistent with our previous observation that OXA‐mediated pre‐synaptic release of GABA in the SCN is critical for OXA's modulatory suppression of SCN neuron activity during the day (Belle *et al*., 2014). Here, we extend these observations to show that this OXA‐GABA‐GABA_A_ receptor‐mediated action also operates to suppress activity in EGFP−ve neurons, eliciting distinct and diverse electrical behaviors in these identified SCN cell populations. Our present results also demonstrate that the magnitude of these OXA actions depends primarily on the intrinsic membrane potential of the neurons.

During the day, the phase of the SCN clock can be reset by so‐called non‐photic/arousal‐promoting stimuli (Mistlberger & Antle, 2011; Hughes & Piggins, 2012). This non‐photic resetting is associated with suppression of SCN cellular activity and clock gene expression (Maywood *et al*., 2002; Hamada *et al*., 2004). One of the main brain structures that communicates non‐photic information to SCN neurons is the thalamic intergeniculate leaflet (IGL) which directly innervates the SCN through the geniculohypothalamic tract (GHT; Harrington, 1997; Golombek & Rosenstein, 2010). An important neurochemical of the IGL‐GHT pathway is neuropeptide‐Y (NPY; reviewed in Yannielli & Harrington, 2004). Indeed, during the day, NPY inhibits SCN electrical activity (including *Per1*‐EGFP cells), suppresses *Per1* expression, and phase‐advances the SCN clock (Liou & Albers, 1991; Medanic & Gillette, 1993; Shibata & Moore, 1993; Huhman & Albers, 1994; van den Pol *et al*., 1996; Cutler *et al*., 1998; Fukuhara *et al*., 2001; Maywood *et al*., 2002; Besing *et al*., 2012; Belle *et al*., 2014).

Surprisingly, the suppressive action of OXA on SCN electrical activity during the day does not have such dramatic effects on clock phase, but instead accentuates the actions of NPY to potently inhibit cellular activity and phase‐shift the SCN clock (Belle *et al*., 2014). These selective effects of OXA may be important in that, when acting alone, it relieves SCN‐inhibition of behavioral activity without affecting overall circadian rhythmicity. This allows animals to respond to acute behavioral necessities, such as grooming and drinking (van Oosterhout *et al*., 2012), without uncoupling behavioral rhythms from prevailing light‐dark cycles. Under circumstances when non‐photic adjustment of the clock phase may be necessary, such as during physiological conditions that promote IGL‐SCN signaling, OXA acts to reinforce these non‐photic signals to the SCN. This implicates OXA signaling in the integration of non‐photic communication in the SCN, and its distinct and diverse effects on the electrical activity of *Per1*‐EGFP+ve and EGFP−ve neurons seen here may partially underpin such capacity and plasticity in its action.

At multiple brain sites OXA acts to cause excitation, but the importance of its engagement with local GABAergic circuits to suppress/modulate spiking activity in cells is becoming more prominent. For example, in the lateral hypothalamus where orexinergic and melanin‐concentrating hormone (MCH) neurons are intermingled, OXA acts through local GABAergic interneurons to mainly suppress electrical activity of MCH cells. This provides a possible switching mechanism which appropriately isolates the conflicting signals of sleep and wakefulness (Apergis‐Schoute *et al*., 2015). Here, and in our previous work, we provide evidence that this local ‘switch control’ also operates in the SCN circuit, which presumably acts in behaving animals to avoid conflicts between circadian and arousal signals (Belle *et al*., 2014). Indeed, our present data provide some insight into the complex nature of such ‘switch control’, indicating that investigation of how this is achieved *in vivo* to govern behavior will be a challenging venture.

Our results indicate that the change in GABAergic PSC amplitude elicited by OXA in *Per1*‐EGFP+ve neurons was significantly larger than those measured in EGFP−ve cells. This likely underpins the significantly greater magnitude of OXA‐evoked RMP suppression in *Per1*‐EGFP+ve neurons than in EGFP−ve cells. As the delta PSC frequency evoked by OXA between these cell types approached but was not statistically significant, the results suggest the existence of mainly clock‐driven post‐synaptic membrane properties/processes that render *Per1*‐EGFP+ve neurons more amenable to OXA‐dependent GABA‐GABA_A_ receptor signaling than EGFP−ve cells. Indeed, even when comparison was made between cell‐types of similar moderate RMPs and firing capability (morning *Per1*‐EGFP+ve vs. EGFP−ve cells), OXA‐induced delta RMP was unexpectedly larger in *Per1*‐EGFP+ve neurons than in EGFP−ve cells. Several intrinsic post‐synaptic membrane properties of neurons are associated with synaptic processing gain, measured as PSC amplitude and summation. In the entorhinal stellate cells, for example, depolarization and increases in input resistance give rise to a voltage‐dependent increase in excitatory and inhibitory post‐synaptic potential amplitude (Economo *et al*., 2014). In a similar fashion, alteration in input resistance can regulate synaptic transmission efficacy at single synapses (see Harnett *et al*., 2012 for example). Increased post‐synaptic receptor density also influences the magnitude of synaptic signaling, by primarily augmenting the PSC amplitude elicited by pre‐synaptic neurotransmitter release, a process that occurs at central GABAergic synapses (Nusser *et al*., 1998). Although the density of post‐synaptic GABA_A_ receptor expression in *Per1*‐EGFP+ve and EGFP−ve neurons is unknown, measurement of membrane properties in these cells revealed that during the day the *R*_input_ and RMP of *Per1*‐EGFP+ve neurons are significantly higher than those measured in EGFP−ve cells (see also Belle *et al*., 2009). This difference in excitability states can therefore account for the distinctive response magnitude to OXA measured in these two cell populations.

Perhaps less surprising is the observation that OXA‐induced delta RMP in morning *Per1*‐EGFP+ve neurons is smaller than that elicited by this neuropeptide in afternoon *Per1*‐EGFP+ve cells. Indeed, as is the case for morning *Per1*‐EGFP+ve and EGFP−ve neurons, on average the intrinsic baseline *R*_input_ value of *Per1*‐EGFP+ve cells is significantly elevated in the afternoon when compared with morning measurements (Belle *et al*., 2009). This positive association of response magnitude with *R*_input_ further reinforces the possibility that post‐synaptic membrane resistance of SCN neurons largely contributes to cell‐state and, to a lesser extent, cell‐type response magnitude to OXA. By extension this post‐synaptic property may also influence the way SCN neurons respond to other neurochemical signals conveyed to this brain structure.

More broadly, our study reveals how intrinsic electrical states can shape neural responses to incoming signals. Hyperexcited and moderately resting neurons with high input resistance are reported in a number of other brain regions, such as the cerebellum, habenula and arcuate nuclei of the mediobasal hypothalamus (Belousov & van den Pol, 1997; Raman *et al*., 2000; Pugh & Raman, 2009; Sakhi *et al*., 2014a,b). As these regions also express circadian clock genes, it is plausible that circadian‐driven neural states determine how input signals, such as those associated with appetite and motor co‐ordination, are received and processed in these brain tissues. Although the function of hyperexcited neurons is yet to be determined, it is likely that, as in the SCN, inhibitory signals conveyed to these brain regions may cause paradoxical generation of spiking activity. Indeed, our manual RMP manipulation in hyperexcited SCN *Per1*‐EGFP+ve cells with steady‐state current injection was able to mimic and recapitulate all of the electrical behaviors elicited by OXA in these afternoon SCN neurons. This demonstrates that by the simple removal of hyperexcitation or depolarization blockade, DLAMOs and normal spiking can be resumed in some of these cells. This argues for a general mechanism through which inhibitory signals, such as OXA acting through GABA, can evoke diverse and complex electrical activities in SCN neurons. Such neuronal state transition by inhibitory signals, as evoked here in the SCN by OXA, likely act to shape how cells respond and process information to subsequent synaptic inputs. For example, the transition from depolarization blockade to spiking would enable cells to be more responsive to subsequent excitatory signals.

The distinct responses to OXA in *Per1*‐EGFP+ve and EGFP−ve cells may therefore be central to the way SCN neurons organize, integrate and appropriately balance incoming excitatory photic vs. suppressive arousal signals in behaving animals across the day. Indeed, photic and non‐photic signals can interact with each other at the level of the SCN; the effects of non‐photic resetting cues are cancelled if the non‐photic signal is followed by a light pulse, or administration of glutamatergic receptor agonists (Biello & Mrosovsky, 1995; Biello *et al*., 1997; Gamble *et al*., 2004). This may permit animals to appropriately respond to potentially competing external and internal signals in order to organize physiology and behavior.

## Author contributions

M.D.C.B. and H.D.P. conception and design of research; M.D.C.B. performed experiments and analyzed data; M.D.C.B and H.D.P. interpreted results of experiments; M.D.C.B, and H.D.P. wrote the manuscript.

## Conflict of interests

The authors declare no competitive financial interests.
